# Evaluation Instruments for Quality of Life Related to Melasma: An Integrative Review

**DOI:** 10.6061/clinics/2018/e65

**Published:** 2018-05-15

**Authors:** Camila Fernandes Pollo, Silmara Meneguin, Helio Amante Miot

**Affiliations:** I Enfermagem, Campus de Botucatu, Faculdade de Medicina, Universidade Estadual Paulista Julio de Mesquita Filho, Botucatu, SP, BR; II Departamento de Dermatologia e Radioterapia, Faculdade de Medicina, Universidade Paulista Julio de Mesquita Filho, Botucatu, SP, BR

**Keywords:** Melasma, Quality of Life, Development, Validation Studies

## Abstract

The aim of this study was to analyze scientific production concerning the validation and cultural adaptation of quality of life evaluation instruments for patients with melasma and to offer a critical reflection on these methods. A literature review was performed based on a search of the Web of Science, Bireme, PubMed, Elsevier Scopus, and Google Scholar databases. All published articles from indexed periodicals in these electronic databases up to December 2015 were included. Eight articles were identified, of which only one (12.5%) referred to the development and validation of a specific instrument for evaluation of the quality of life of melasma patients. An additional six articles (75%) referred to transcultural adjustment and validation of the same instrument in other languages, and another (12.5%) article reported the development of a generic instrument for evaluation of quality of life in patients with pigment disorders. This review revealed only one specific instrument developed and validated in different cultures. Despite being widely used, this instrument did not follow the classic construction steps for psychometric instruments, which paves the way for future studies to develop novel instruments.

## INTRODUCTION

Melasma is a recurrent chronic change in pigmentation characterized by asymptomatic, symmetrical, hyperpigmented macules in the skin due to local hypermelanogenesis that affects millions of people worldwide 1.

Melasma mainly affects women of fertile age in photoexposed areas, especially the face 2. Hispanic and Asiatic populations have a higher incidence 3, as do populations living in areas with strong solar radiation, such as India, Pakistan, the Middle East, and the Mediterranean 4.

The high level of population miscegenation in Brazil and its predominantly tropical climate favor the development of the disease in the country. When considering the different regions and their ethnic compositions, the authors estimate that between 15 and 35% of adult Brazilian women are affected by melasma 5.

Although few population studies in Brazil have evaluated the prevalence of the disease, pigmented dermatosis is the third most common cause of consultations in dermatology clinics (8.4%), with variation based on the age group, gender and region of the country 6. For instance, the prevalence of melasma was 22% in a study with adults in public institutions from inland Brazil 5.

Treatment of melisma as a chronic disease results in the use of only palliative treatments. Because it only occurs in visible areas, melasma can have a significant impact on the quality of life. However, quality of life is a subjective concept linked to the perspective of each individual.

Psychometric questionnaires for the evaluation of health-related quality of life (HRQoL) can be divided into two types (generic and specific). The generic instruments are multidimensional and are developed to evaluate the impact caused by a disease in various aspects of life. The disadvantage of these instruments is that they are not sensitive for the detection of specific aspects of the impact on the quality of life inflicted by a particular disease 7.

Specific instruments can also be multidimensional and can be used to evaluate particular aspects of the quality of life of a specific individual, with an emphasis on symptoms, incapacities, or limitations related to a specific ailment. The disadvantage of these instruments is the difficulty in understanding the general phenomenon 7.

In this context, despite the relevance of evaluating quality of life in patients with melasma for clinical practice and treatment, no recent studies have permitted integration through reviews of the measurement instruments available in the literature.

Health assistance providers in Brazil use a single translated and validated instrument, which does not always incorporate regional specifics. This investigation was instigated in view of the need to investigate the most used instruments to evaluate the quality of life in patients with melasma and to contribute to the development of future research for nursing in this area.

## OBJECTIVE

The objective of this study was to understand and analyze scientific production related to quality of life evaluation instruments for patients with melasma.

## METHOD

An integrative review study was performed using the following steps: identification of the hypothesis or guiding question, followed by a search using descriptors or keywords; establishing inclusion and exclusion criteria for sample selection; study categorization; evaluation of studies; discussion and interpretation of the results; presentation of the integrative review and knowledge synthesis 8.

To guide an integrative review, the following question was formulated: “How many instruments are available in the literature to evaluate quality of life in patients with melasma?”

The inclusion criteria for this integrative review were as follows: articles published before December 31, 2015; articles indexed as validation studies in the study databases; and articles published in Portuguese, English, or Spanish.

Articles were identified in the Web of Science, Bireme, PubMed (PMC), Scopus, and Google Scholar databases using the keywords melasma, quality of life, development and validation studies through search strategies using the AND Boolean operator.

The information was summarized and organized using an instrument constructed for this purpose by taking into consideration the following aspects: title; author; periodical; year of publication; instrument used to evaluate the quality of life; database; sample; validity; and internal consistency (Cronbach’s alpha).

The results were presented, and the obtained data were discussed descriptively to reach the objective of this method.

After reading and analyzing the studies, we opted to group them into two categories as follows: specific instruments to evaluate the quality of life related to melasma (QoLRM) and specific instruments to evaluate the QoLRM validated and adapted to other cultures.

## RESULTS

**Figure 1:**
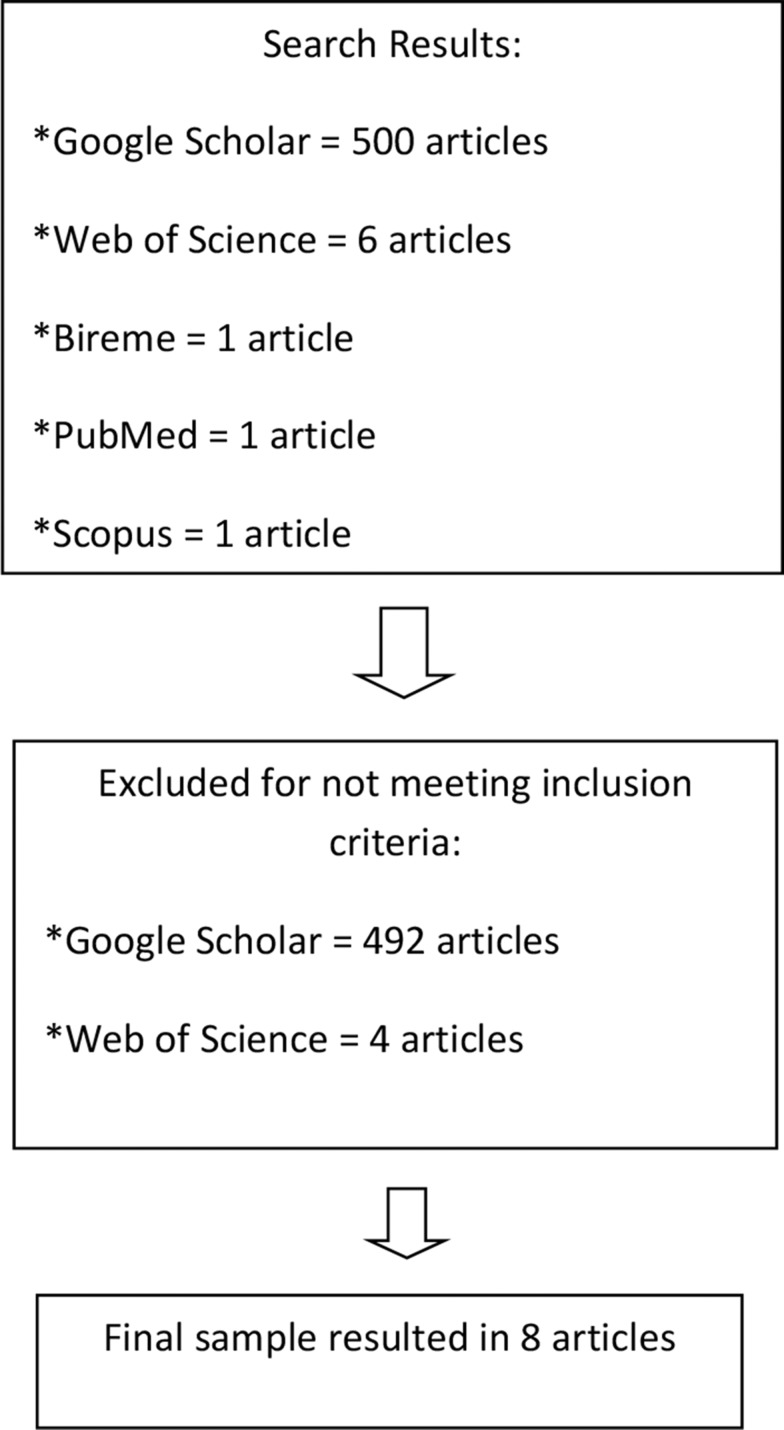
Flow diagram representing the article selection process.

The search identified 509 articles. A total of 501 articles were excluded after reading the abstracts, because they did not meet the established criteria. In other words, they did not address cultural and language development and adaptation. Thus, the final sample consisted of eight articles, as shown in Figure 1.

All of the articles were configured as methodological investigation studies, in which transverse delineation predominated to meet the proposed objectives.

The articles were published during the period between 1999 and 2014, as shown in Table 1.

The studies were performed in the United States (12.5%), South America (37.5%), Western Europe (25%), and Asia (25%). The majority of the periodicals were in the area of dermatology, with a mean impact factor of 3.6 (1.76-5.0).

In addition to the specific instrument used to evaluate the quality of life in melasma patients (MelasQoL), two articles (25%) concomitantly used the WHO Quality of Life – Brief (WHOQOL-Bref), and one article (12.5%) used one a non-cited Spanish questionnaire on the quality of life. Two other articles (25%) used specific instruments to evaluate the quality of life in dermatology, and three articles (37.5%) did not refer to the use of any other instrument.

### Specific instrument to evaluate the quality of life related to melasma (QoLRM)

**Figure 2:**
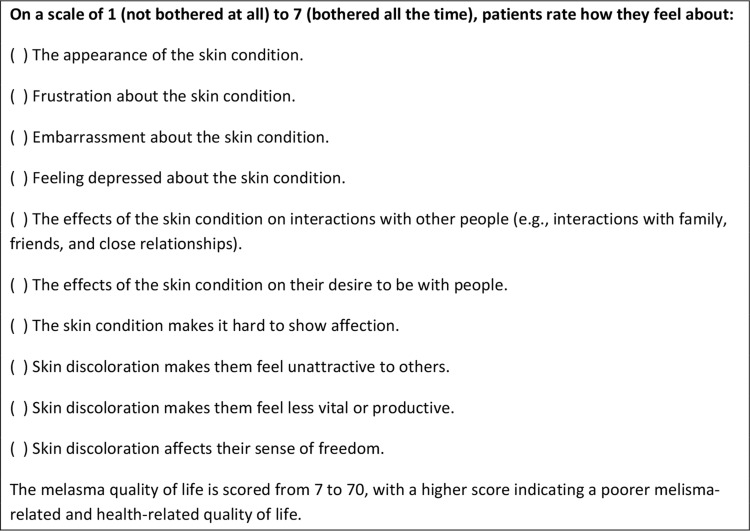
MelasQoL Questionnaire.

This review only identified one specific instrument for evaluation of the QoLRM. In 2003, a researcher at the University of Virginia developed and validated the MelasQoL (Melasma Quality of Life Scale) 9, which is an instrument that specifically contains ten items to evaluate the effect of melasma on the emotional state, social relationships, and daily activities. This instrument was developed from compiling seven questions from the SKINDEX-16 questionnaire (Quality of Life Index related to skin diseases) and three items from an unspecified questionnaire about changes in skin color. Thus, the instrument was not based on classical psychometric steps or the symbolic perceptions of the patients (Figure 2).

The English version of the MelasQoL displayed high internal consistency, validity, and discriminatory power compared to the general questionnaires for evaluating quality of life in dermatology [the DLQI (Dermatology Life Quality Index) 10 and SKINDEX-16 11]. Although the authors state that the MelaQoL is one dimensional, statistical data show that this instrument is multidimensional.

From this perspective, the MelasQoL is a valid and reliable instrument that can be used to evaluate the repercussions of melasma on the patient’s quality of life.

### Specific instrument to evaluate the QoLRM validated and adapted to other cultures

Transcultural adaptation of the original questionnaire into Spanish 12 was performed using directives established in the literature. The pre-test was performed on a group of 30 patients and then applied to 112 sequential patients at a community outpatient clinic. The level of melasma was determined by clinical examination using the Melasma Area and Severity Index (MASI). Other authors have concluded that this instrument is a semantic translation equivalent to the original English version of the MelasQoL.

Validation of the Brazilian Portuguese version of the MelasQoL 13 was performed using a multicenter study with 300 individuals from five different Brazilian geographical regions. The analysis revealed an important impact of the disease in relation to skin appearance, since 65% of the participants referred to being uncomfortable with blemishes all or the majority of the time, 55% felt frustrated, and 57% were ill at ease due to the condition of their skin.

This study concomitantly used both the MASI and the World Health Organization instrument for quality of life evaluation (WHOQOL-BREF) to respectively evaluate the melasma severity and quality of life. The instrument displayed significant internal consistency (Cronbach’s alpha=0.92; *p*<0.01) and good correlation with the MASI scores. The results of this study demonstrated that the MelasQoL-BP was a valid instrument that could be used to evaluate quality of life in Brazilian patients with melasma 13.

The validation process for the Turkish version of the MelasQoL (MelasQoL-Tr) 14 included 114 melasma patients. The MASI, MelasQoL-Tr, and WHOQOL-BREF were usedto evaluate the melasma severity and quality of life. The reliability and validity of the MelasQoL-Tr demonstrated that this instrument was valid and reliable for evaluation of the quality of life in Turkish patients with melasma.

During the translation and validation process for the Arabic version 15, 65 female patients participated in the study using the MASI. Internal consistency was tested for all ten items on the MelasQoL scale (Arabic version) with excellent results. An intra-class coefficient of correlation (ICC) of 0.914 and a Cronbach’s alpha of 0.94 indicated high reliability for the Arabic version. A positive correlation was found between the MelasQoL scale and MASI scores (r=0.41). The analysis revealed that the Arabic version of the MelasQoL scale was a reliable and valid measure for evaluation of the quality of life in Arabic patients with melasma.

The validation performed in Colombia included 80 women and obtained the following validation criteria: internal consistency with Cronbach=0.88; validity with rs=0.70, *p*<0.001; and reproducibility with intra-class coefficient of correlation=0.959 (CI 95%: 0.986, *p*<0.001), the main conclusion was that the scale was a good indicator of the quality of life in the studied group of Colombian women with melasma 16.

Finally, all of the above cited instruments began with the translation and cultural adaptation of the original MelasQoL 9 instrument, and the objective of the authors was to develop and validate a specific instrument for the disease to identify the dimensions of life most affected by melasma.

The other instruments identified in the articles and used together in the studies, such as the DLQI (Dermatology Life Quality Index) and SKINDEX-16, were not included in the analysis, because they were considered specific instruments for evaluating the quality of life in general dermatology. Some authors also used generic instruments, such as the SF-12 and WHOQOL.

Table 2 summarizes the publications related to the validation and transcultural adaptation of the MelasQoL.

## DISCUSSION

Although asymptomatic, melasma affects exposed areas, such as the face, mainly in women of fertile age, which maximizes its impact on body image and interpersonal relationships and thus potentially interferes with the perception of quality of life.

The literature has shown the need to look more deeply at patients with dermatological problems that are often neglected as not being life threatening 17. In dermatology, health professionals must evaluate the quality of life of those affected by skin pigmentation disorders, because many complaints and problems can be resolved or treated when individuals feel that they are listened to, understood, and respected 18.

The results of this study identified only one specific instrument in the literature for evaluation of the quality of life in patients with melasma (MelasQoL); this instrument has been translated and validated for other cultures. However, the development of this instrument did not follow the classic steps of psychometry for the construction and validation of a psychometric instrument, since the selection process was based on the composition of other questionnaires and not on the individual symbolic perceptions of the patients.

One point is that this instrument stimulates questioning related to instrument reliability, even though the original version in English presented high internal consistency, validity, and discriminatory power compared to other questionnaires. Another point to be considered is that the validation process has only been applied in women.

Additionally, the latent factorial structure and dimensionality of the MelasQoL have not been adequately explored in development and validation studies. At this stage, no studies have examined construct stability (test-retest) 9.

Furthermore, the high level of item subjectivity and the number of reply options stimulate criticism regarding possible difficulty in understanding by the patients, the dissociative behavior of item scores from a lack of evaluation of the impact of the disease in personal relationships and the low correlation with clinical scores, which could render the results poorly representative. Strikingly, the instrument does not rank the impact on the quality of life or even categorize the severity based on score behavior. The temporal stability of the questionnaire was not measured, and the dimensionality was not adequately explored 9.

Another interesting point is that the MelasQoL uses a small number of items to represent psychological aspects linked to melasma compared to its impact on social relationships, leisure, profession, and physical appearance. Despite the simplicity and applicability of an instrument with only ten items, representation of the measurement of feelings and perceptions linked to self-esteem was less important to authors, which potentially compromised instrument precision. Furthermore, items related to emotional wellbeing have been identified as having a high magnitude in quality of life studies on melasma 19-21.

This review identified only one specific instrument for evaluation of the quality of life in patients with melasma, which was developed and validated for different cultures. However, despite being very widespread, the instrument did not follow the classic construction stages for psychometric instruments, which supported the premise of its fragility. The other selected articles were related to the translation and validation processes to other cultures.

This study contributes to the field, because it shows a gap in this area of understanding and paves the way for the development and validation of other instruments for evaluating quality of life related to melasma.

## AUTHOR CONTRIBUTIONS

Pollo CF was responsible for the manuscript critical review. Meneguin S and Miot HA provided orientation and critically reviewed the manuscript.

## Figures and Tables

**Table 1:** List of the articles included in this literature review. Botucatu, SP, Brazil, 2015.

N	Title	Author	Periodical Year	Instruments	Database
1	Development and validation of a quality of life measurement for chronic skin disorders in French	Grob JJ, Auquier P, Martin S, Lançon C, Bonerandi JJ.	Dermatology / 1999	SF-36 VQ-Dermato	Google Scholar
2	Development and validation of a health-related quality of life instrument for women with melasma	Balkrishnan R, McMichael AJ, Camacho FT, Saltzberg F, Housman TS, Grummer S, et al.	Br J Dermatol/ 2003	MelasQoL Skindex-16 DLQI Skin discoloration questionnaire	PubMed/Scopus Web of Science Bireme Google Scholar
3	Validation of a melasma quality of life questionnaire for Brazilian Portuguese language: the MelasQoL-BP study and improvement of QoL of melasma patients after triple combination therapy	Cestari TF, Hexsel D, Viegas ML, Azulay L, Hassun K, Almeida AR, et al.	Br J Dermatol/ 2006	MelasQoL WHOQOL-BREF MASI	Google Scholar
4	Melasma in Latina patients: cross-cultural adaptation and validation of a quality-of-life questionnaire in Spanish	Dominguez AR, Balkrishnan R, Ellsey AR, Pandya AG.	J Am Acad Dermatol/ 2006	MelasQoL Instrument for quality of life in Spanish MASI	Google Scholar
5	Translation and cultural adaptation to Portuguese of a quality of life questionnaire for patients with melasma	Cestari TF, Balkrishann R, Weber MB, Prati C, Menegon DB, Mazzotti NG, et al.	Med Cutan Iber Lat Am/ 2006	MelasQoL Not cited	Google Scholar
6	Validation of a melasma quality of life questionnaire for the Turkish language: the MelasQoL-TR study	Dogramaci AC, Havlucu DY, Inandi T, Balkrishann R.	J Dermatolog Treat. / 2009	MelasQoL WHOQOL-BREF MASI	Google Scholar
7	Validation of the MELASQoL quality of life index in a group of Colombian melasma patients	Gómez LJ	Repository.urosario.edu.co	MelasQoL Not cited	Google Scholar
8	Reliability and validity of the Arabic version of the Melasma Quality of Life questionnaire: MELASQoL-A) study	Abou-Taleb DA, Youssef EM, Ibrahim AK, Moubasher AE.	Acta Derm Venereol / 2014	MelasQoL Not cited MASI	Google Scholar

**Table 2:** Publications in relation to validation and transcultural adaptation works of MelasQoL. Botucatu, SP, Brazil, 2015.

N	Country	Sample	Internal Consistency Cronbach’s Alpha	Reliability
1	United States	102	0.95	The MelasQoL scores were highly correlated with the other QoLRS measurements, highlighting that the instrument was reliable.
2	Brazil	147	0.89	Reliability analysis revealed a satisfactory result.
3	Brazil	300	0.91	Analysis of MelasQoL-BP demonstrated the disease had an important impact on the QoL.
4	Spain	112	0.91	No significant differences were found between the domain coefficients. Many of these QoLRS domains were identified as significantly affected by melasma.
5	Turkey	114	0.88	Correlation of the MelasQoL-Tr with the WhoQoL-Bref and MASI demonstrated that the instrument was reliable.
6	Arabia	65	0.94	Internal Consistency for the 10 items in the Arabic version of the MELASQoL-A questionnaire was excellent; ICC=0.914 and Cronbach’s alpha=0.944, indicating high reliability.
7	Colombia	80	0.88	ICC=0.959, obtaining good indices for evaluating the quality of life in Colombian women.
